# Social and Poor-Law Problems

**Published:** 1908-02-08

**Authors:** 


### SOCIAL AND POOR-LAW PROBLEMS.

### THE UNEMPLOYED.

It is winter, and the cry of unemployment is again heard. It is about twenty years since the unemployed began to bulk largely in the national view. There were trade conditions which then justified people in assuming that the trouble was a temporary one, and temporary measures were taken to relieve distress. How futile these Mansion House funds and similar efforts were it is not necessary to recall. Their one useful result has been the attempt to put the relief of the unemployed on a more satisfactory and reasonable basis. What was once local, unorganised, spasmodic, is now national, organised, and permanent. The effort has been made not to lessen the energy of voluntary charity, and yet to guide it to sensible results. But everywhere we are met with the assurance that the result is mere failure, and that distress is as rampant as ever. If, whether trade be good or bad, this question of the unemployed is to be ever with us, we are justified in inquiring why this should be so.

#### The Question of Character.

To this question many sections of society will give answers modified by their personal experience and predilections, but there are two points which cannot be ignored—the character of the unemployed, and the kind of employment they want. Character in this connection does not necessarily mean bad character. A man may be a good man, thoroughly honest and reliable, a good husband and father, and yet be out of work. For if, with all these qualities, he is physically weak, or if he is unintelligent, or if he is slow, he may be turned off as soon as the pressure of work which made his employer glad of any assistance, however poor, is over. In business moral qualities are valuable adjuncts to physical, intellectual, and technical merits, but they do not take their place. This fact is known to many who, on the strength of the moral qualities of a man, have tried to employ him.

#### Willingness.

Next comes the question of what a man is willing to do. The out-of-work whom you accost will assuredly say that he will do anything to gain an honest living. It is to meet the case of these that what is called test-work has been devised. The task set demands a moderate degree of physical strength

THE UNEMPLOYED—*continued.*

and comparatively little intelligence. It can be done by the average of men, and payment is proportioned not to the work done, but to the needs of the worker and his family. As a matter of fact the work is done, and done easily, by the average loafer. It would seem then that an unemployed man would gladly seize this opportunity of showing that he was really willing to do anything he could to earn an honest living. Some do, but one finds a large proportion who say that this test-work is "degrading."

### THE DEGRADATION OF DOING TEST JOBS.

The "degradation" consists, one is told, in the fact that the work has no economic value. It is not useful work; it is only test-work. Then these same individuals say that they went employment at their various trades. When one considers the variety of trades represented by the unemployed, the foolishness of the demand becomes apparent. It is practically impossible to provide work for every man who happens to be out of employment. By work we mean work for which there is a real industrial demand. If that demand is non-existent, the "work," whatever its nature, is as futile, and therefore degrading, as any breaking of stones in a stone-yard can be. If utility is called in as a test of honourableness, those who have invoked it have no excuse for objecting to the verdict. If out of the ratepayers' money, or out of voluntary charity we set the bricklayer to build houses which no one needs, or the carpenter to make tables and chairs which no one wants to buy, we may be letting them down gently and saving their feelings, but we are not giving them "work" in any economic sense of the term. Work means something that is wanted; and on those who claim the "right to work" devolves the responsibility of finding the want which they can fulfil. Certainly on this point a Government department can do good work. For the genuine "out-of-work" who desires to find employment it is a great matter to know where his special skill and knowledge can be best employed. The knowledge being given him, let him go where he is wanted, and it would be no great misuse of voluntary funds to pay the cost of his journey.

### SENTIMENTAL CONSIDERATIONS.

But here, as all who have tried to help this class will admit, sentimental considerations step in. Our "out-of-work" will not leave his wife and family, he will not break up his home to go elsewhere. We have known one unemployed whose spirit of chivalry was so high that he refused to go to Canada (where work had been promised him) until he (or charitable friends) liquidated the debts he had contracted here. It had not hurt his conscience to contract the debts. It would not hurt his conscience to go on trusting to luck or charity for the continued existence of himself and his family, but he would not go abroad with his debts unpaid! The man was not dishonest; he was merely stupid. And this makes one great difficulty in dealing with the unemployed. They are generally the most unintelligent of the working classes. They have learned one or two socialist catchwords, which they employ without much knowledge of their meaning. This "right to work" is one of them. And they interpret it to mean the opportunity of working at any trade they know in the environment which is familiar to them. And if "work" of this kind cannot be brought to them, they claim the right to be kept. And by that they mean also to be kept in the circumstances and in the environment to which they are accustomed. To tell the truth, one could hardly lower their standard of life, the comforts to which they are accustomed are but few. But as soon as one offers these comforts, or even greater under circumstances which are unpleasant to accept, there is an outcry of inhumanity, brutality, breaking up the home, and what not. It is again a matter of the test, and there will always be people who will resent any test being applied.

### INDISCRIMINATE CHARITY.

Yet when charity gives to an unknown entity—as it does in most cases—it will always demand some kind of a test, not of worthiness, but of sincerity. If the applicant will not meet this demand we are justified in assuming that his poverty is less than his pride, and perhaps that his energy is less than either. If he cannot, or will not, accept work on the terms on which it can be had—for this is no age of economic tyranny, but one in which everyone wants to help the willing worker—we are justified in assuming that what is sought is not work, but maintenance. And, indeed, that is what one is forced to believe in with many of the unemployed.

### THE AGE LIMIT.

Looking at those doleful processions that parade our streets through the winter, the first thing that one notes is the number of old men. Now when a man whose sole stock-in-trade was his physical strength gets old, his usefulness diminishes. He is bound to give way to younger and stronger men. He becomes "unemployed," and except for periods of pressure, which are few and uncertain, will be "unemployed" for the rest of his life. No one can give him real economic "work," for he has no longer strength, and he never had much training or intelligence. There remains, then, charity—either the charity to which, as a citizen, he has a right, that is, the Poor-law, or private charity. The same law holds good of even the trained workman in a dying industry, especially if he is too old or too conservative to try to change his trade. When ill-health or dissipation is the cause of unemployment the result is still more inevitable, for with these no change is possible. While, therefore, these causes remain it is idle to expect that unemployment will vanish from among us. Those who think that under any circumstances it can are either ignorant or too idealistic for this world. But among the unemployed there will always be a number who are willing to submit to due tests, to make reasonable experiments to raise themselves from their unhappy condition. For the sake of these any effort to help the mass is justifiable, and by the result in these cases is it justified, even though in the others it may hopelessly fail.

